# Feasibility of a theory-based intervention towards benzodiazepine deprescribing in Belgian nursing homes: protocol of the END-IT NH cluster-randomised controlled trial

**DOI:** 10.1136/bmjopen-2024-085435

**Published:** 2024-10-22

**Authors:** Perrine Evrard, Tina Chevallereau, Josephine Aikpitanyi, Catherine Pétein, Sandy Tubeuf, Séverine Henrard, Anne Spinewine

**Affiliations:** 1 Clinical Pharmacy and Pharmacoepidemiology Research Group, Louvain Drug Research Institute, UCLouvain, Brussels, Belgium; 2 Institute of Health and Society (IRSS), UCLouvain, Brussels, Belgium; 3 CHU UCL Namur, Pharmacy department, UCLouvain, Yvoir, Belgium

**Keywords:** Aged, Drug Utilization, Feasibility Studies, Implementation Science, Quality Improvement

## Abstract

**Introduction:**

Despite several calls to deprescribe benzodiazepine receptor agonists (BZRA) in older adults, their use among nursing home residents (NHRs) remains high. Therefore, we developed an intervention targeting general practitioners’ and healthcare professionals’ behaviours regarding BZRA deprescribing in nursing homes (NHs): The END-IT NH (bENzodiazepines Deprescribing InTerventions Nursing homes) 6-component intervention. Before moving on to a large-scale effectiveness and cost-effectiveness evaluation, this feasibility study aims at: (1) assessing the feasibility of the intervention implementation in NHs, (2) assessing the feasibility of conducting a larger-scale evaluation, in terms of recruitment and data collection and (3) conducting an exploratory cost-effectiveness evaluation.

**Methods and analysis:**

We will conduct a cluster-randomised controlled trial in a sample of 6 NHs, with 10–15 NHRs included per NHs. Four NHs will be randomised into the intervention group, and two NHs will deliver usual care (control group). Data collection will occur at baseline, 3, and 6 months (study end). We will collect information to explore implementation fidelity, mechanisms of impact and contextual factors at patient-level, NH-level and healthcare professional-level, using both quantitative and qualitative measures. The feasibility of the study conduction will be assessed by measuring recruitment and attrition rates and completeness of data collection. An exploratory cost-effectiveness evaluation will be conducted based on quality of life and healthcare use and cost data.

**Ethics and dissemination:**

This study protocol received approval from the ethical committee of CHU UCL Namur on the 20 June 2023. All data are confidential and will be anonymised prior to analysis. De-identified data will be shared on a data depository with a 2-year embargo. The results of the study will be disseminated through a scientific paper and will be communicated to local stakeholders and policymakers through a local symposium.

**Trial registration number:**

NCT05929443.

**Strengths and limitations of this study**

- This study evaluates the feasibility of an intervention that has been developed with insights from implementation science and stakeholders’ involvement.
- Having this feasibility study stage will enhance the probability of success of the intervention in a future larger-scale trial and is likely to save resources.
- This feasibility study encompasses different dimensions of feasibility, at intervention and study design levels.
- Nursing homes (NHs) will be recruited voluntarily, and this may select NHs with extra motivation for benzodiazepine receptor agonists deprescribing.

## Introduction

 Benzodiazepine receptor agonists (BZRA, namely benzodiazepines and Z-drugs) are authorised medicines in the treatment of severe anxiety or short-term treatment of insomnia. BZRA are commonly used among Belgian nursing home residents (NHRs), with a prevalence of use of 52.4%.^1 2^ However, these medicines have an unfavourable benefits-risk profile in the older population.^3^ Indeed, BZRA only have modest benefits^4^ while they can trigger severe side effects including inappropriate sedation, increased risk of falls and fractures, cognitive impairment and dependence.^35^,^7^ Consequently, BZRA use should be avoided in older adults or limited to 4 weeks (2 weeks for insomnia).^8^,^10^ Alternatives exist, such as non-pharmacological approaches or utilisation of antidepressant in the case of severe anxiety.^11^

Deprescribing is the action to discontinue or reduce the use of a medication that is no longer needed or that may put the patient at risk, under the supervision of healthcare professionals (HCPs), with the goal of managing polypharmacy and improving outcomes.^12^ In terms of deprescribing in older adults, BZRA are considered priority medicines.^13^ BZRA can be safely deprescribed in nursing homes (NHs), via slow tapering and dose reduction.^14^ Still, current deprescribing rates are low,^1^ and many barriers to deprescribing remain. Data from two systematic reviews^15 16^ indicate that some barriers are specific to the context and setting of care, while others are more generic, such as lack of knowledge and skills among HCPs and patients, perceptions of BZRA effectiveness and fear of withdrawal symptoms. An intervention targeting identified barriers is expected to enhance BZRA deprescribing.

Many BZRA deprescribing interventions have already been studied—mainly in the outpatient setting.^17^ These interventions encompass medication review, substitution, educational programmes or mixed interventions. They achieved BZRA deprescribing rates ranging from 27% to 80%.^17 18^ However, it is unclear which components of interventions are the most effective, and data from the NH setting remain scarce. Context is a key component that can influence the effect of the intervention and is significantly different between the NH and the ambulatory setting.^15 19^ Additionally, previously developed interventions for medication optimisation in NHs often lacked theoretical background,^20^ which is in contradiction with intervention development recommendations.^21^ These insights align with a broader call to leverage implementation science in order to increase the translation of deprescribing evidence into practice.^22 23^ In light of this, the present research programme aims at developing and evaluating a theory-based intervention that would target general practitioners (GPs) and HCPs behavioural determinants and promote BZRA deprescribing, in the specific context of Belgian NHs. The avenues we are exploring include the careful identification of implementation strategies, a focused attention on contextual determinants and the evaluation of implementation outcomes.

The present study is part of an overall research programme that follows the French 4-step model of theory-based intervention development,^24^ and recommendations of the Medical Research Council (MRC) on developing and evaluating complex interventions.^21^

We developed an intervention towards BZRA deprescribing in NHs, using a theory-based process and with the involvement of appropriate stakeholders (HCPs involved in NH care, NH direction, policymakers and residents or relatives). The behaviours targeted are, for (1) GPs, to initiate BZRA deprescribing discussion with NHRs and relatives, and initiate deprescribing when indicated, as part of patient care, and in collaboration with other HCPs, and for (2) other HCPs, to support GPs and NHRs in BZRA deprescribing. Briefly, we first identified barriers and enablers for BZRA deprescribing by performing a systematic review followed by a qualitative study, and mapping barriers and enablers with the Theoretical Domains Framework (TDF).^15 25 26^ Using theory in intervention development is recommended by the MRC framework. Theory may help in understanding the factors influencing the targeted behaviour, in identifying possible techniques to promote the behaviour change^27^ or in developing the programme theory of the intervention (how the intervention is supposed to have its effect, and under which conditions^21^). The TDF was chosen as a theoretical framework, as it specifically aims at identifying influences on HCPs’ behaviour related to the implementation of evidence-based practices or intervention.^28^ The intervention was then developed to target the nine TDF domains that we identified as BZRA deprescribing determinants in Belgian NHs: Knowledge, skills, social professional role and identity, beliefs about capabilities, beliefs about consequences, goals, memory attention and decision process, environmental context and resources and social influences. We worked with stakeholders to select acceptable, practicable, effective, affordable, safe and equitable behaviour change techniques (BCTs) from the BCT taxonomy V.1,^29^ with evidence of links to these TDF domains.^30^ Selected BCTs were then operationalised in a 6-component intervention. Intervention development and content have been described in detail elsewhere,^31^ and a summary of the intervention is available in the methods section. The programme theory is available in online supplemental appendix 1 and summarises the targeted behaviour, TDF domains, intervention components, potential mechanisms of impact and expected behavioural and long-term outcomes.

The present study now aims at evaluating the feasibility of the END-IT NH (bENzodiazepines Deprescribing InTerventions Nursing Home) intervention. Feasibility testing is recommended by the MRC and aims to evaluate the feasibility and acceptability of both the intervention and its evaluation design. Based on the feasibility study results, a decision should be made on whether to go on with the evaluation or not. The intervention can also be refined before the evaluation, and the programme theory adapted.^21^ Feasibility studies increase the chance of success of the following main studies, and they provide useful preliminary data that can be used by other researchers when developing future study designs.^32^

The objectives of the present feasibility study are threefold:

1. To evaluate the feasibility of implementing the END-IT NH intervention in Belgian NHs.
2. To assess the feasibility of conducting a larger cluster randomised controlled trial to evaluate the effectiveness and cost-effectiveness of the END-IT NH intervention, considering the many challenges of conducting a deprescribing trial in the NH setting.
3. Provided that the feasibility of the needed data collection is validated, to explore the potential cost-effectiveness of the intervention.

## Methods

The protocol of the END-IT NH feasibility study (V.1, on date of 25 May 2023) has been registered on ClinicalTrial.gov. The Standard Protocol Items: Recommendations for Interventional Trials (SPIRIT) checklist recommendations for interventional trials^33^ and the Consolidated Standards of Reporting Trials checklist extension to randomised pilot and feasibility trials^34^ guided the reporting of this protocol. See online supplemental appendix 2 for the SPIRIT checklist.

### Study design, setting and participants

We will conduct a cluster-randomised, parallel-group, open-label controlled trial, with NHs being the clusters. As this feasibility study does not aim at evaluating the effectiveness of the END-IT NH intervention for BZRA deprescribing, sample size calculation was not computed. Six NHs will be included. In each of these NHs, 10–15 NHRs will be included. The study will take place between July 2023 and March 2024.

Four of the six NHs will be randomised to the intervention group, and two to the control group. The higher number of NHs in the intervention group will help inform our primary research objective, which is evaluating the feasibility of implementing the END-IT NH intervention. Randomisation of NHs will be performed after the recruitment of NHRs and baseline data collection. Because of the small number of clusters, randomisation will not be stratified. The randomisation will be computerised and done through Research Electronic Data Capture (REDCap). This step will be performed by a member of the research team involved neither in the recruitment nor in the data collection. Because of the nature of the intervention, blinding will not be feasible.

An overview of the study design and timeline is available in online supplemental appendix 3.

### Recruitment process and eligibility criteria

#### Nursing homes

NHs were recruited through previous collaborations and mailing of the official list of existing NHs in Wallonia (French-speaking part of the country). Interested NHs were invited to an online meeting during which the research project was presented, and NHs were asked to confirm interest. Six NHs were then selected, with the aim of maximising diversity in regards to the type of ownership, localisation (rural vs urban) and presence of a specific Alzheimer unit.

Participating NHs will sign a convention with UCLouvain, defining the responsibilities of the NHs, modalities of compensation, recruitment and data collection. Each NHs will receive a financial compensation for their time participating in the study: €40 per NHR for recruitment and data collection. Intervention group NHs will additionally receive €1500 for implementing the intervention. The repartition of this compensation will be left to the NH’s discretion.

#### Nursing home residents

To be eligible, NHRs must be aged 65 years and older and be taking at least one benzodiazepine or Z-drug for 4 weeks or longer. Exclusion criteria are: inability to communicate in French, palliative care, ongoing BZRA or alcohol withdrawal, severe anxiety and severe depression (as clinically judged by GPs and/or nurses). These exclusion criteria were selected to avoid including NHRs for which BZRA deprescribing would not be recommended. The NH staff will establish a list of eligible residents and will approach eligible NHRs using an information letter. Written informed consent will be asked by physicians (GP or coordinating physician (CP)) or nurses to each resident or to a resident’s representative if the resident is not mentally competent (eg, in case of severe dementia). For residents who are not able to read or write in French, an impartial witness will assist in the recruitment process. The resident will give consent orally, and the witness will sign the informed consent. No financial compensation will be granted to NHRs for their participation.

### Intervention

**Figure 1:**
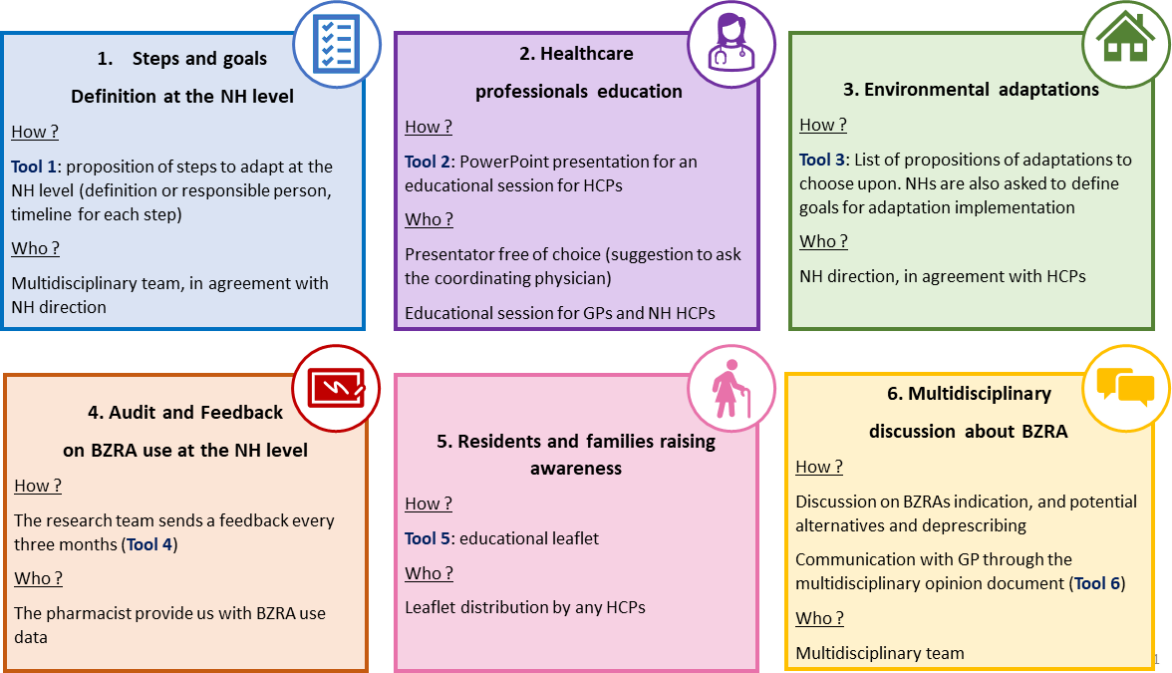
Summary of the intervention components. BZRA, benzodiazepine receptor agonists; GP, general practitioner; HCP, healthcare professional; NH, nursing home.

NH allocated to the intervention group will implement the END-IT NH intervention. The six intervention components are summarised in figure 1 and described below. A more detailed description is available elsewhere.^31^

1. Process and goals setting: NHs direction and HCPs will be asked to define steps in BZRA deprescribing, and to set goals reachable at the level of their NH. For this purpose, we developed a list of possible steps and goals. This tool has been developed to be operationalised at the level of the NH, meaning that each NH can adapt the steps, add steps, appoint a person responsible for them and a timeline.
2. HCPs education: We will provide material for HCPs training in a PowerPoint format. This training encompasses information about BZRA side effects and deprescribing process. Additionally, it prompts HCPs to reflect on the pros and cons of deprescribing, and on case vignettes. The training session will be organised at the level of each NH, preferably by the CP, whose role includes staff training.
3. Physical environment adaptation: We will provide NHs with a list of possible adaptations to promote relaxation and sleep, and each NH will be able to choose what adaptations are feasible in their specific context. This tool allows NHs to appoint responsible staff for adaptations and due date. Adaptations beyond those listed in the document are also possible.
4. Audit and feedback: At the beginning of the intervention and every 3 months, we will provide NHs with feedback data on BZRA use in their NH. This feedback will be based on the percentage of residents taking at least one BZRA (ATC codes N05CD, N05CF, N05BA or N03AE01), as reported by the NH pharmacist. This percentage will be compared with the percentage of NHRs taking at least one BZRA in a previous study conducted in 54 Belgian NHs.^1 2^
5. NHRs and their relatives’ awareness: We will provide an educational leaflet, adapted from the EMPOWER brochure, which has already been shown to be effective in BZRA deprescribing.^35 36^ The adaptation for the Belgian and NH context was performed with the involvement of appropriate stakeholders (HCPs, older adults, NHR) (Pétein *et al*^37^).^31^ This leaflet encompasses information about BZRA side effects, non-pharmacological alternatives for sleep issues and anxiety, a testimony of an NHR and an example of an 18-week tapering scheme. NH staff and/or GPs will be in charge of distributing the leaflet to NHRs and/or relatives. NH staff may choose how and when to use this brochure depending on their specific context. For example, the brochure may be used to introduce deprescribing with the NHR, or HCPs may choose to distribute the brochure to all NHRs they think might benefit from it. In the context of residents with dementia, it is expected that relatives will be more involved.
6. Multidisciplinary work: Our intervention will promote multidisciplinary review of BZRA indications and opportunities for deprescribing. We developed a multidisciplinary opinion document to enable communication between NH staff and GPs, if GPs do not attend multidisciplinary meetings. This tool enables NH staff to report potential BZRA side effects to the GP, and to suggest changes in prescriptions or non-pharmacological approaches. The GP can respond on the same document. As for other intervention components, the NH will have flexibility in how they organise the multidisciplinary meetings and use the multidisciplinary opinion document.

### Control

NHs in the control group will keep working as usual. After the 6-month data collection, they will have access to the intervention material.

### Measures

#### Feasibility of intervention implementation

Our plan to evaluate the feasibility of the intervention follows the approach for process evaluations recommended by the MRC guidance.^38^ We will collect information to better explore implementation fidelity, mechanisms of impact and contextual factors at patient-level, NH-level and HCP-level, using both quantitative and qualitative measures. First, we will assess implementation fidelity that refers to the degree to which an intervention or a programme is delivered as intended.^39^ According to the conceptual framework for implementation fidelity developed by Carroll *et al*, two essential aspects will be measured: **adherence** which is ‘how far those responsible for delivering an intervention actually adhere to the intervention as it is outlined by the developers’ and **participants’ responsiveness** to the intervention which is ‘how far participants respond to, or are engaged by, an intervention’.^39^ Second, we will evaluate the mechanisms of impact, that is, how the effects of the intervention occurred. And finally, we will assess how different elements of context can influence participant’s responsiveness and adherence to the intervention as well as mechanisms of impact.

In addition to the quantitative data collection (see table 1), two semi-structured interview guides, with open-ended questions and predefined major themes will be discussed with residents and HCPs. These themes have been identified to investigate the TDF domains targeted by the intervention. All interviews will be audio-taped, and the verbatim will be transcribed before analyses.

**Table 1:** Overview of quantitative data collected and time schedule

Measure	Data collected/instrument for data collection	Data source	Data collection point		
			Baseline	3 mo	6 mo
**Feasibility of intervention implementation**					
Implementation fidelity					
Adherence at the NH level	Organisation of educational sessions for HCPs; number of environmental adaptations implemented; number of residents’ brochures distributed; number of included residents for whom BZRA was discussed in multidisciplinary meetings; global number of implemented intervention components (0–6).	Intervention logs filled by NH staff			X
HCPs’ responsiveness	Attendance to educational and multidisciplinary meetings; number of multidisciplinary opinions sent to the GP and responded to by GP.	Intervention logs filled by NH staff			X
NHRs’ responsiveness	Number of BZRA deprescribing attempts; reasons for not attempting BZRA deprescribing; number of BZRA deprescribing success (including dose reduction and complete cessation, and failure); reasons for deprescribing failure; switch to another sedative or anxiolytic medication.	Intervention logs filled by NH staff			X
Mechanisms of impact					
NHR level	NHRs’ self-efficacy regarding BZRA deprescribing (medication reduction self-efficacy scale, one item); knowledge regarding BZRA (five items); intention regarding BZRA deprescribing (two global items of the rPATD-BZRA questionnaire).	NHR†	X	X	X
NH level	Quality of interdisciplinary collaboration (16 items, adapted from Orchad *et al*^55^).	HCPs	X	X	X
HCP level	HCPs’ perceived knowledge (four items), skills (one item), beliefs about capability (three items) and prioritisation (two items) regarding BZRA deprescribing (Shapoval *et al*, in preparation).	HCPs	X	X	X
Contextual factors					
NHR-level	Quality of life: EuroQol 5-Dimension Questionnaire (EQ-5D-5L)*^56^; length of BZRA intake; main reason for BZRA intake; number of previous deprescribing attempt.	NHR or relative	X		
	Diagnostic of dementia.	MR	X		
NH-level	Type of NH ownership; presence of a specific Alzheimer’s unit; use of automatised dispensing system; existence of a collaboration between the NH and a geriatric team.	NH staff	X		
	Quality of interdisciplinary collaboration (16 items evaluated on a 5-points Likert scale).	HCPs	X		
Feasibility of study design					
Recruitment process	Time for NHR recruitment.	NH staff	X		
	Rate of NHRs’ study participation acceptation.	NH staff	X		
Attrition rate	Rate of participants lost to follow-up.	/			X
Data collection	Rate of missing data for NHR data.	/			X
NH satisfaction with study process	Satisfaction survey, (10 items, adapted from Smailes *et al*^40^).	NH staff			X
Additional NHR-centred outcome					
Socio-demographic data	Age, gender, educational level, time since NH entry, MRS/MRPA status.	NHR or relative, and MR	X		
Quality of life	EQ-5D-5L.*^56^	NHR or relative	X	X	X
Quality of sleep	Insomnia Severity Index.*^57^	NHR†	X	X	X
Anxiety	Geriatric anxiety inventory – short form.*^58^	NHR†	X	X	X
Patient’s attitudes towards BZRA deprescribing	rPATD-BZRA questionnaire.*^59^	NHR†	X		X
Comorbidities	History of dementia, delirium, fall.	MR	X	X	X
Medication use	Number of regular medications, BZRA, other psychotropics (ATC codes N05, N06 and N02), and over-the-counter sleep or anxiety medication use (specialty used, dosage, frequency of use).	MR	X	X	X
Health-economic evaluation					
Sleep and anxiety medication cost	BZRA and BZRA alternatives use and cost.	MR	X	X	X
Healthcare use and available costs	Number of visits to the general practitioner, a specialist, a psychologist, or the emergency department; any hospitalisation (and length of stays); falls.	MR		X	X
Intervention cost	Financial compensation granted to the NHs; budget allocated to the environmental adaptations in the NH.	Intervention logs filled by NH staff			X

MRS/MRPA status stands for ‘“Maison de repos et de soin », or « Maison de repos pour personnes âgées »’, in French. This reflects the level of dependency and of care need of the resident.

* Copyright license and rights were obtained to use these questionnaires.

† Data not collected in residents with dementia.

BZRA, benzodiazepine receptor agonist; GP, general practitioner; HCP, healthcare provider; MR, medical record; NH, nursing home; NHR, nursing home residentrPATDrevised patients’ attitudes towards deprescribing

#### Feasibility of the study design

The feasibility of the study design will be evaluated through four aspects detailed below. First, while recruiting residents, NHs will be asked to record the age and sex of approached residents, and whether the patient agrees, disagrees or gives no answer regarding potential participation in the trial. These records will allow the estimation of the recruitment rate and of differences between participants and non-participants. NHs will also have to evaluate the necessary time for recruitment. Second, the retention of participants will be assessed at each of the data collection points. We will record the number of study withdrawals along with the reasons for withdrawal, patients lost to follow-up and deaths. Third, data will be collected in both study arms as they would be in a full implementation trial in order to assess the rate of missing data. Finally, we will also ask NHs study coordinators to complete a satisfaction survey, regarding the study process. This survey has been adapted from Smailes *et al*.^40^

#### Exploratory cost-effectiveness evaluation

A specified economic component will evaluate the feasibility of collecting data on resource use, health and non-health-related benefits. More specifically, it will check the completeness and the ability to obtain data on quality of life using the EuroQol 5-Dimension Questionnaire and healthcare use from patients in the target population to allow estimation of healthcare costs.^41^ Information on resource use will be obtained at 3 months and 6 months and combined with reference prices to measure the total healthcare costs for the intervention and control groups. The costs of medications will be estimated afterward based on the average national drug cost.

### Data collection and management

#### Quantitative data

Three quantitative data collection points are planned: baseline, 3 months, and 6 months (end of the study). The types of data collected, and the timeline are summarised in table 1.

All data will be collected by one member of the research team, or by a study nurse/job student trained by the research team, at the site of the NH. Data on NHRs will be collected from the medical record and also directly from residents, using questionnaires. Proxy data will be collected from relatives of residents with dementia. Because all questionnaires do not have a version available for relatives, fewer data will be collected on residents with dementia (see table 1 for details). Additionally, we will collect implementation data from intervention logs, and from questionnaires completed by HCPs and NHs study coordinators. For HCPs questionnaire, in all participating NH, the NH study coordinator will recruit four to seven HCPs. At minimum, we will ask for the coordinating physician, another GP and two nurses to participate. These HCPs will give oral consent for participation.

All collected quantitative data will be collected and managed using REDCap electronic data capture tools hosted at UCLouvain.^42 43^ REDCap (Research Electronic Data Capture) is a secure, web-based software platform designed to support data capture for research studies.

#### Qualitative data

Qualitative data will be collected by a trained psychologist, at the end of the study, through interviews with HCPs and NHRs from the intervention group. We will aim at interviewing two NHRs and four HCPs of each intervention NH. Purposive sampling will be used to ensure a good diversity representation of residents and HCPs included in the study. As an example, we plan on including residents with a long or short history of BZRA intake, and residents with and without successful deprescribing. Cognitively impaired NHRs unable to take part in a 45 min interview will not be eligible, but their relative could be interviewed if relevant. Eligible HCPs will be approached by our research team and NHRs by HCPs to ask whether they would be willing to take part in a one-to-one 45 min interview. NHRs participating in the qualitative study will have to provide a specific written informed consent. HCPs will provide oral consent.

### Statistical analysis

Quantitative data will be reported using descriptive statistics. Categorical variables will be expressed as numbers and percentages and continuous variables as mean±SD or median (P25–P75) depending on normality assessment. Χ^2^ test (or Fisher’s exact test), Student’s t-test or Mann-Whitney test will be performed to assess differences between intervention and control group regarding patients’ characteristics. Assessments will not be blinded. This feasibility study will not be powered to assess the effectiveness of the intervention. BZRA cessation at 6 months as well as BZRA dose reduction will be reported in each group, but no statistical evaluation will be performed on any primary or secondary outcome measure. Lost to follow-up patients or withdrawn patients will be classified with patients that would have failed BZRA deprescribing.

All qualitative interview transcripts will be entered into QSR NVivo V.11 for data analysis. Inductive and deductive thematic analysis will be used to analyse transcripts content. Interviews will mainly be coded into the TDF domains explored in the interview guides. However, new themes or subthemes may be identified inductively from the data. Two coders will individually analyse the first two interviews for NHRs and for HCPs. They will meet to compare their coding. If satisfying agreement is reached, the remaining interviews will be coded by a single coder. If not, another two interviews will be coded by both coders independently, and coding will again be compared. The above-mentioned method will be used until reaching satisfying agreement, ensuring results reliability and validity.

Missing data will inform the research team of the feasibility of data collection. Hence, the relevance of some variables that would have too many missing values will be discussed prior a potential full implementation trial.

Regarding the health-economic evaluation, provided that the feasibility of the needed data collection is validated, we will conduct an exploratory cost-effectiveness analysis on completed cases following the recommendations of the Belgian Healthcare Knowledge Centre.^44^ The combination of answers to EuroQol 5-Dimension will lead to a health profile of five digits that will be converted into a utility using standard Belgian tariff values.^45^ It will estimate patients’ quality-adjusted life years (QALYs) and associated costs. Given the findings from the analysis, we will conduct a value of information analysis to determine whether future research is worthwhile to assess cost-effectiveness.^46^ We will also calculate the incremental cost per QALY gain to deprescribing BZRA at 6 months compared with usual care by dividing the average difference in cost by the average difference in QALYs to generate the incremental cost-effectiveness ratio (ICER). The ICER represents the additional cost per one unit of outcome gained and indicates the trade-off between total cost and effectiveness when choosing between deprescribing BZRA and usual care.^41^

All quantitative analyses will be performed using R software (‘R Foundation for Statistical Computing, Vienna, Austria’).

### Patient and public involvement

NHRs and relatives representatives were involved in the development of the END-IT NH intervention, from the identification of barriers and enablers for BZRA deprescribing to the operationalisation of BCTs into intervention components. Results will be discussed with an advisory board that will include older adults’ and carers’ representatives in addition to HCPs and policymakers. This advisory board will help make decisions on the opportunity of refining the intervention components and proceeding to a full implementation trial. This advisory board will also be involved in results dissemination.

### Ethics and dissemination

This study protocol has been submitted to the ethical committee of CHU UCL Namur and received approval on the 20 June 2023 (NUB: B0392023000052).

All data are confidential. Data will only be available to researchers involved in this study and they will be pseudo-anonymised prior to analysis. To do so, a coded number will be attributed to every participant. The correspondence between the coded number and participant’s name will be kept in a separate numerical file, protected with a password and destroyed after 7 years. Following the FAIR Data Principles (or Findable, Accessible, Interoperable, Reusable) recommend data sharing,^47^ de-identified data will be shared on a data depository (OSF^48^) with a period of embargo of 2 years. The DOI of the dataset will be communicated to the scientific journal to which we will submit the results of this study.

Results of the study will be disseminated through a scientific paper and will be communicated to local stakeholders and policymakers through a local symposium, along with resulting recommendations.

## Discussion

This study will evaluate the feasibility of implementing and evaluating our theory-based intervention towards BZRA deprescribing, through a cluster-randomised controlled trial study design. To our knowledge, this is the first study evaluating an intervention specifically developed towards BZRA deprescribing, in French-speaking Belgian NHs. Based on the results of this study, a decision will be made to move forward or not with a larger-scale intervention effectiveness evaluation. The intervention itself and its methods of delivery might be adapted based on the implementation results, before moving on to the larger scale study. Study design and outcome measures may also evolve, depending on the results relative to study conduction and data collection.

We anticipate difficulties regarding recruitment and data collection process with NHRs. Indeed, older adults’ recruitment in trials is a challenge that can be impacted by various factors.^49 50^ A recent study reported that the three main reasons for older adults to decline participating in a deprescribing trial are (1) feeling overwhelmed by current health status, (2) lack of interest or mistrust of research and (3) hesitancy to participate in a deprescribing study.^51^ Some of these barriers might be enhanced in the NH setting. The recruitment phase has been reported as the most difficult part of research in NH. NHRs often fail to see how they would benefit from research participation, and obtaining consent from cognitively impaired NHRs or their relatives also appears as a difficulty.^52^ We chose to have the recruitment process done by HCPs involved in the daily care of the NHRs (GPs or nurses), which might reduce NHRs’ mistrust. Furthermore, our informed consent form does not mention the word ‘deprescribing’ but refers to ‘reconsidering hypnotics and anxiolytics use’. This will also reduce the risk of recruiting only residents in favour of deprescribing. Regarding the data collection, several outcomes require long questionnaires to be filled with NHRs. Even if these questionnaires have generally been developed for older adults, and because NHRs are generally frailer than the average older adults, we expect that the length of the questionnaires might be an issue. Therefore, depending on the evaluated quality of collected data, questionnaires used might evolve before a larger-scale study. Additionally, to enhance study retention, in case of NHR tiredness, we will shorten data collected with NHRs and only collect data on quality of life.

Recently, Thorpe *et al* developed a framework of factors/data that should be taken into consideration when designing an observational deprescribing trial in NHs, using routinely accessible data.^53^ In the Belgian NH context, such routine data are not available. Still, it is interesting to compare the factors they suggest exploring to what we have planned in our prospective design. According to their framework, the following factors should be explored as deprescribing determinants: Intrapersonal factors (socio-demographic characteristics, condition and medication attributes, prognosis and co-prescribed medications), interpersonal factors (family caregiver level of engagement, and HCPs predisposition to deprescribing), organisational and health system factors (facility resources, care coordination), community factors (geographical patterns of healthcare use and deprescribing) and policy factors.^53^ Most of the suggested factors will also be explored in our study. Geographical patterns of the NHs are not collected as such, but we will aim to involve NHs with variation in this regard. Finally, the impact of policies is being studied separately, as part of a broader project on deprescribing. Still, this was evaluated in our qualitative preparative work,^25^ and might be addressed in the qualitative process evaluation.

This study has several strengths. First, a feasibility study stage is likely to save resources by avoiding engaging in an unfeasible full trial and will enhance the probability of success of the intervention in a future full trial. Second, the design of the trial is as pragmatic as possible, and the intervention implementation is flexible, to enable variations regarding each NH specificities. Third, the intervention and its methods of delivery were developed with insights from theory and stakeholders’ involvement, which should enhance its implementation and effectiveness. Finally, the intervention will be implemented at the level of the NH, and the randomisation will be clustered, to avoid contamination bias.

This study also has limitations. First, because of the nature of the intervention, blinding of participants will not be feasible. Researchers responsible for data collection will also be aware of group allocation. To minimise the impact of this limitation, the baseline data collection will be performed in each site before randomisation. Second, participating NHs are recruited on a voluntary basis and these may have higher awareness and motivation regarding BZRA (de)prescribing than other Belgian NHs. Third, some of the exclusion criteria are based on GPs’ and nurses’ clinical judgement and not on validated scales. Fourth, as the intervention is complex and includes six components, it will not be possible to know which intervention component is responsible for its effect. However, the mixed-method process evaluation will help us understand the impact of the intervention on potential mechanisms of impact. Fifth, potential harms and assessment of causality are not explicitly foreseen in this feasibility trial, but should be addressed in a full trial. Sixth, our intervention is implemented at the NH level, meaning that NHRs not included in the trial might be exposed to it. However, for ethical reasons we are not able to assess the effects of the intervention on these NHRs. Finally, we did not predefine progression criteria to decide whether to go on with the evaluation in a full trial. Progression criteria, recommended by the MRC framework, ‘help researchers interpret their pilot trial findings to decide whether, and how, to proceed with a future definitive trial’.^54^ Defining these progression criteria would have been difficult, as the intervention is a novel combination of components, and its feasibility will partly be evaluated through a qualitative study. Therefore, the decision to move on with a full effectiveness trial will be made by the research team, without a priori defined progression criteria, but taking into consideration recommendations from the advisory board.

### Trial status

First patients were recruited in July 2023. The 6 months quantitative data collection is currently ongoing and planned to end in March 2024. The qualitative part of this study will begin in March 2024.

## supplementary material

10.1136/bmjopen-2024-085435 online supplemental file 1

10.1136/bmjopen-2024-085435 online supplemental file 2

10.1136/bmjopen-2024-085435 online supplemental file 3
