# Supplementary material for: Feasibility of a theory-based intervention towards benzodiazepine deprescribing in Belgian nursing homes: protocol of the END-IT NH cluster-randomised controlled trial
Source: BMJ Open. 2024 Oct 22;14(10):e085435. doi: 10.1136/bmjopen-2024-085435 (PMC11499836; doi:10.1136/bmjopen-2024-085435)
Supplement: online supplemental file 1 [file bmjopen-14-10-s001.pdf]

Problem to address

BZRA have an unfavourable benefits-to-risk ratio among older adults and should be limited to a 4-weeks use. Guidelines recommend deprescribing chronic BZRA.

Despite that, BZRA use in Belgian NHs remains high (52,4% Evrard et al, 2020), and 98% of users take BZRA for more than 4 weeks.

This shows a poor uptake of BZRA deprescribing in Belgian NHs.

Behaviours

Who: GP
What: Deprescribe (the process of stopping the use or reducing the dosage) BZRA, through slow tapering
Where: in NH
When: During a medical health consult at the NH
To/with whom: Patients of the GP institutionalized in a NH and under BZRA prescription, involved through shared-decision making,

Who: HCPs involved in multidisciplinary NH care
What: Support the deprescribing process of BZRA in the institution, with two levels involved:
- Support to the GP
- Support to NHR and their family

Where: in NH
When: As part of NHRs' daily care
To/with whom: Action taken for NHRs, involving various HCPs.

Influences on behaviour

Knowledge*

Skills

Social/ Professional rôle and identity*

Beliefs about capabilities

Beliefs about consequences*

Memory, attention and decision process

Goals

Environmental context and resources

Social influences

Intervention components [and BCTs]

Process and Goals setting
[Graded tasks]
[Goals setting (behavior)]

HCPs education
[Instructions on how to perform the behaviour]
[Information about health consequences]
[Problems solving]
[Pros and cons]

Physical environment adapations
[Restructuring physical environment]

Audit and feedback
[Social comparison]

NHRs and relatives education
[Instructions on how to perform deprescribing]
[Information about health consequences]
[Pros and cons]

Multidisciplinary work
[Restructuring social environment]

Intervention processes

-Increased interdisciplinary collaboration

-Increased HCPs' perceived knowledge regarding BZRA

-Increased HCPs' beliefs about capability for BZRA deprescribing

-Increased HCPs' priorisation of BZRA deprescribing

-More sleep-friendly environment

- Decrease in social influences of NHRs reluctance towards deprescribing (:Increased NHRs' self-efficacy regarding BZRA deprescribing, Increased NHRs' knowledge regarding BZRA deprescribing, Increased NHRs' intention for BZRA deprescribing)

Behavioural outcomes

-Multidisciplinary discussion on potential BZRA deprescribing and non-pharmacological alternatives

-HCPs discussion with NHRs about BZRA use and potential deprescribing

- BZRA tapering plans prescribed

Longer term outcomes

-More appropriate use of BZRA in NHs

-Lower prevalence of BZRA use without substitution to other medications (e.g. antidepressants, antipsychotics)

-Increased NHRs safety (reduction in falls, dependence, oversedation, increased mobility, increased daytime functioning…)

Appendix 1: Programme theory of the END-IT NH intervention

Notes: BCT = Behaviour change technique, **BZRA** = Benzodiazepine Receptor Agonists, **GP** = General practitioner, **HCP** = Healthcare professional, **NH** = Nursing home, **NHR** = Nursing home resident

*: These domains were identified as most relevant only for HCPs other than general practitioners
