# Supplementary material for: Feasibility of a theory-based intervention towards benzodiazepine deprescribing in Belgian nursing homes: protocol of the END-IT NH cluster-randomised controlled trial
Source: BMJ Open. 2024 Oct 22;14(10):e085435. doi: 10.1136/bmjopen-2024-085435 (PMC11499836; doi:10.1136/bmjopen-2024-085435)
Supplement: online supplemental file 3 [file bmjopen-14-10-s003.pdf]

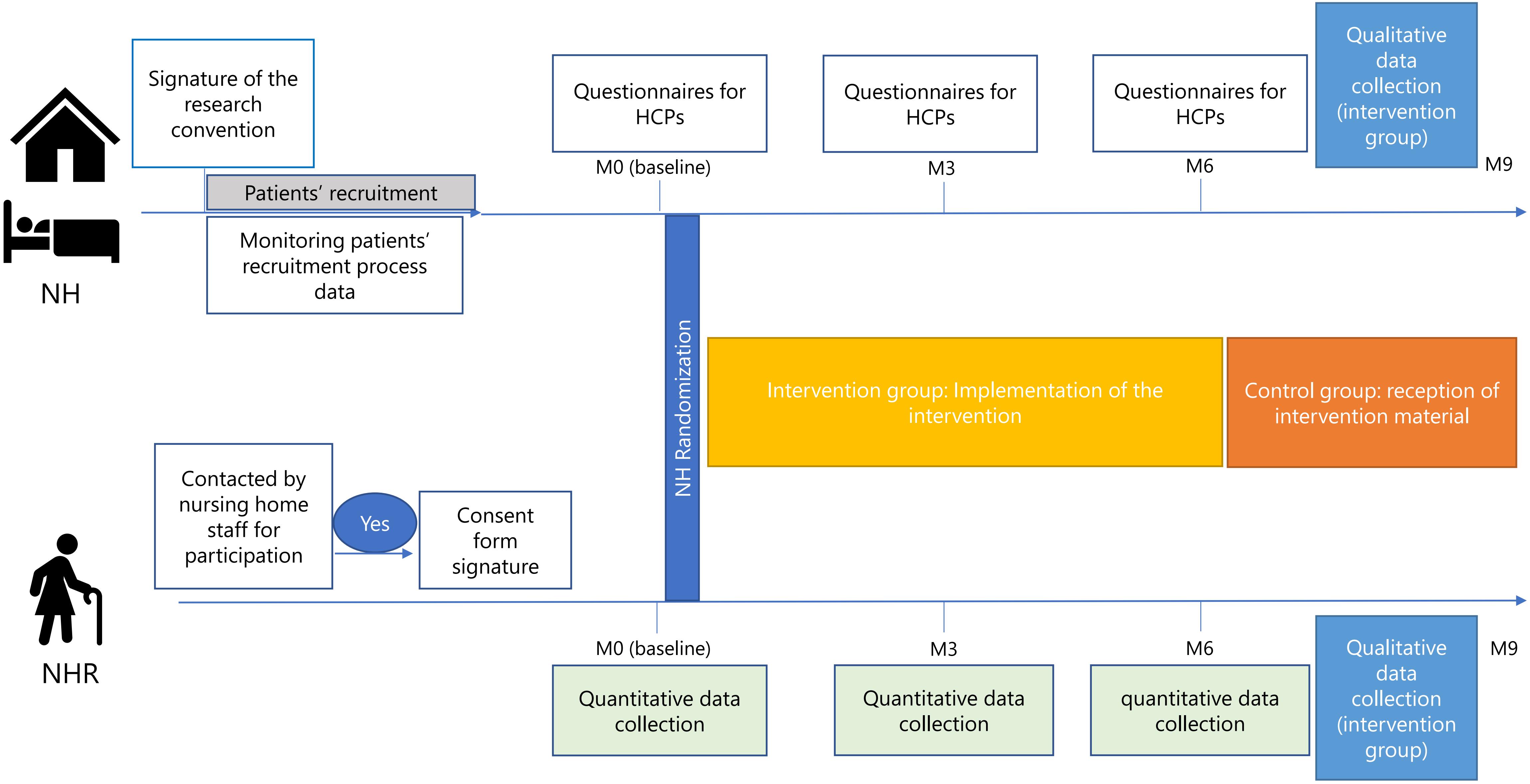


**Appendix 3:** Study timeline: Recruitment and data collection

**Legend: HCP=** Healthcare professional, **M=** month, **NH=** nursing home, **NHR=** nursing home resident. Month 0 data collection was considered study baseline
